# Additional considerations before using a ctDNA-guided approach for informing adjuvant chemotherapy in colorectal cancer

**DOI:** 10.1186/s12916-023-03037-9

**Published:** 2023-09-08

**Authors:** Timothée Olivier, Alyson Haslam, Vinay Prasad

**Affiliations:** 1 Department of Oncology, Geneva University Hospital, 4 Gabrielle-Perret-Gentil Street, 1205 Geneva, Switzerland; 2 Department of Epidemiology and Biostatistics, University of California San Francisco, 550 16th St, 2nd Fl, San Francisco, CA 94158 USA

**Keywords:** Adjuvant therapy, Oncology, Molecular test, Colon cancer, ctDNA, Evidence-based medicine, Non-inferiority

## Abstract

**Background:**

The DYNAMIC trial investigated the use of circulating tumor DNA (ctDNA) to guide adjuvant treatment decisions in stage II colon cancer. Despite the DYNAMIC trial’s assertion that a ctDNA-guided approach could minimize the use of adjuvant treatment without compromising recurrence-free survival (RFS), we raised concerns regarding the trial’s methodology and the practical implications of its findings in a Debate article. Here, we expand upon these concerns in a response to a correspondence by the authors of the DYNAMIC trial.

**Main body:**

We dispute the choice of a large non-inferiority margin in the DYNAMIC trial, simply because an 8.5 percentage points decrease in recurrence-free survival could result in significant harm to patients. We challenge the authors’ comparisons of the DYNAMIC trial outcomes with observational studies. Such comparison is subject to selection bias and changes over time that limit their relevance. The prognostic role of ctDNA do not automatically imply that more treatment in patients with ctDNA positivity would improve outcomes, which we highlight. In real-world settings, we anticipate a potential rise in chemotherapy use due to clinicians utilizing ctDNA alongside existing clinicopathologic factors, rather than using ctDNA as an entire replacement. Lastly, a key concern in DYNAMIC was an 350% higher use of oxaliplatin in the ctDNA arm compared with standard management (9.5% versus 2.7%, respectively), which poses a risk for long-term neuropathy.

**Conclusion:**

We look forward improvements in patient selection in the adjuvant setting, but we maintain our reservations about the DYNAMIC trial and the real-life implementation of its results. As an alternative to exploring de-escalation strategies with large margins non-inferiority trials, we propose that superiority trials in stage II patients could be a more effective strategy.

## Reply

In their recent Correspondence, Gibbs et al. [1] commented on our Debate article ‘Molecular testing to deliver personalized chemotherapy recommendations: risking over and undertreatment.’ Our Debate article argued that molecular testing with the aim of personalizing chemotherapy risked both under- and over-treatment. One of our arguments focused on the DYNAMIC trial, which was authored by Gibbs and colleagues.

As a background, in the DYNAMIC trial, patients with stage II colon cancer were randomly assigned (2:1) to either a ctDNA-guided approach or a standard approach to inform adjuvant treatment decision. The trial aimed to demonstrate that using a ctDNA-guided approach would result in a reduction in the use of adjuvant treatment without negatively affecting the risk of recurrence. The trial was a non-inferiority trial, and its primary endpoint was to show non-inferiority in recurrence-free survival at 2 years. The results showed that the ctDNA-guided approach was non-inferior to the standard approach, with a recurrence-free survival rate of 93.5% in the ctDNA group and 92.4% in the standard management group. Moreover, a lower proportion of patients in the ctDNA-guided group received adjuvant chemotherapy compared to the standard management group (15% versus 28%).

The goal of medical therapy is to maximize survival and quality-of-life, and all else being equal, to use as little treatment as possible in this goal. In this regard, the DYNAMIC trial suffered from key limitations which we raised in our work [2]. Here, we offer several counter-points to those raised in Gibbs et al.’ correspondence [1].

### Non-inferiority margin and positive study

The aim of a non-inferiority trial design is to demonstrate that a new treatment or strategy is not inferior to an established treatment by a pre-specified margin, known as the non-inferiority margin. However, an issue commonly encountered in non-inferiority trials is the choice of margin width, which refers to how much loss of effectiveness is deemed acceptable [3]. Here, Gibbs et al. claim DYNAMIC is a positive study [1]. This fails to address the concern that the non-inferiority margin—8.5 percentage points—is large. An 8.5 percentage points loss in disease-free survival (DFS) in stage II colon cancer is detrimental, even with the theoretical benefit of less treatment for patients. When a large margin is chosen, a positive trial is almost predestined [3].

### Limitation in observational data comparison

Comparing randomized-controlled trial results to population-based comparative efficacy research performed using observational studies is subject to biases, mainly because of selection bias and changes over time. A lack of significant correlation between the survival hazard ratio estimates reported by observational studies and randomized trials has been previously shown [4]. Gibbs et al. write: “The high 2-year RFS seen in treated ctDNA positive patients is in stark contrast to the very high recurrence rate in observational series” [1]. Comparing subgroup results from a highly selected population like in the DYNAMIC trial to historical data provides limited information, especially in light of the significant changes and advancements in this field over the past decades, which we have highlighted in our original work [2]. It is impossible to separate the benefit of the strategy from secular trends and selection bias.

### Difference between prognostic and predictive factors

Prognostic factors are used to predict the natural course of a disease, while predictive factors are used to predict response to treatment. Gibbs et al. mention that “there is arguably under treatment, as recurrences do occur in stage II colon cancer that were not treated” [1]. We disagree with their interpretation: simply because recurrences occur does not mean a treatment will automatically improve the outcome. It remains to be shown whether ctDNA technology can identify a subset of stage II colon cancer patients who derive net benefit from chemotherapy.

### Unintended consequences of the use of ctDNA in real-world practice

We contend that if ctDNA is made broadly available in community practice, clinicians will naturally use it in addition to current clinicopathologic characteristics and not necessarily in lieu of them. Given that the design of DYNAMIC does not allow for physicians to prescribe comparably to real-world practices, the real-life implication may result in much more people receiving chemotherapy, as we demonstrated in our original work [2]. This is simply because high-risk patients identified by the ctDNA will be included with patients previously identified by the standard clinicopathologic approach.

### Modification of margins post-hoc

Pre-specified statistical plans aimed to prevent multiple analyses occurring after the results are seen, p-hacking, and other questionable research practices. Gibbs et al. claimed the DYNAMIC trial “would still have been positive even if a much tighter margin had been used” [1]. We disagree with this interpretation of the DYNAMIC trial, as pre-registered statistical designs are meant to avoid such unreliable conclusion.

### Higher oxaliplatin use and risk of neuropathy

In DYNAMIC, the intervention arm sought to minimize the use of chemotherapy, but rates of oxaliplatin use were 350% higher in the ctDNA arm compared with standard management (9.5% versus 2.7%, respectively). Oxaliplatin is the most onerous portion of adjuvant chemotherapy primarily because its use poses a risk for long-term neuropathy. Therefore, a strategy resulting in more oxaliplatin use without proof of improved DFS may actually be a harmful strategy. Gibbs et al. did not address this primary concern of the DYNAMIC trial [1].

### Superiority trials as an alternative

Instead of exploring de-escalation strategies with non-inferiority trials with large margins, we posited that superiority trials might be better designed to identify subgroups benefiting from chemotherapy among stage II patients which are not treated with chemotherapy according to current management (there was a typo in our original essay, which mentioned stage III). This point remains valid.

## Conclusion

We eagerly look forward to improved patient selection in the adjuvant setting, but our concerns about the DYNAMIC trial and the real-life implementation of its results persist.

## Data Availability

All data on which this work was based are publicly available.

## Abbreviations

ctDNA: Circulating tumor DNA
DFS: Disease-free survival
RFS: Relapse-free survival
